# Smurfs in Protein Homeostasis, Signaling, and Cancer

**DOI:** 10.3389/fonc.2018.00295

**Published:** 2018-08-02

**Authors:** Praveen Koganti, Gal Levy-Cohen, Michael Blank

**Affiliations:** Laboratory of Molecular and Cellular Cancer Biology, Azrieli Faculty of Medicine, Bar-Ilan University, Safed, Israel

**Keywords:** Smurf1, Smurf2, ubiquitination, protein degradation, cell signaling, cancer

## Abstract

Protein ubiquitination is an evolutionary conserved highly-orchestrated enzymatic cascade essential for normal cellular functions and homeostasis maintenance. This pathway relies on a defined set of cellular enzymes, among them, substrate-specific E3 ubiquitin ligases (E3s). These ligases are the most critical players, as they define the spatiotemporal nature of ubiquitination and confer specificity to this cascade. Smurf1 and Smurf2 (Smurfs) are the C2-WW-HECT-domain E3 ubiquitin ligases, which recently emerged as important determinants of pivotal cellular processes. These processes include cell proliferation and differentiation, chromatin organization and dynamics, DNA damage response and genomic integrity maintenance, gene expression, cell stemness, migration, and invasion. All these processes are intimately connected and profoundly altered in cancer. Initially, Smurf proteins were identified as negative regulators of the bone morphogenetic protein (BMP) and the transforming growth factor beta (TGF-β) signaling pathways. However, recent studies have extended the scope of Smurfs' biological functions beyond the BMP/TGF-β signaling regulation. Here, we provide a critical literature overview and updates on the regulatory roles of Smurfs in molecular and cell biology, with an emphasis on cancer. We also highlight the studies demonstrating the impact of Smurf proteins on tumor cell sensitivity to anticancer therapies. Further in-depth analyses of Smurfs' biological functions and influences on molecular pathways could provide novel therapeutic targets and paradigms for cancer diagnosis and treatment.

## Introduction

Protein ubiquitination is a major posttranslational modification that controls a wide spectrum of biological functions, and is critical in maintaining cellular homeostasis under physiological conditions and in diseases.

Ubiquitination is a multi-step enzymatic process which is mediated by the concerted action of three main types of proteins: (i) ubiquitin-activating enzymes (E1s), which bind, adenylate and activate cognate ubiquitin molecules using the energy of ATP hydrolysis; (ii) ubiquitin-conjugating enzymes (E2s), which accept ubiquitin from E1 in the form of a thioester bond to their active-site cysteine; and (iii) ubiquitin protein ligases (E3s) that recruit ubiquitin-charged E2 enzymes and mediate specific transfers of ubiquitin to protein substrates.

It is estimated that the human genome encodes for more than 630 E3s, ~40 E2s, and only two E1s. E3 ubiquitin ligases are of particular interest since they define the spatio-temporal nature of ubiquitination and, together with other accessory proteins, provide specificity to the cascade.

E3s tightly control protein stability, localization, and function, and thereby regulate a plethora of biological processes. This has instigated intensive investigations of these enzymes as disease biomarkers and drug targets in a variety of human disorders, particularly in cancer (1–3).

Depending on the ubiquitin transfer mechanism and domain characteristics, E3s are classified into three main groups/families: really interesting new gene (RING) family, which is the most abundant in the human genome (~600 family members), homologous to the E6AP carboxyl terminus (HECT) domain E3s (~30 members), and RING-in-between-RING (RBR) E3s (~12 in humans) (4).

Smurf1 and Smurf2 (Smurfs) are two closely related C2-WW-HECT domain E3 ubiquitin ligases, belonging to the NEDD4 subfamily of HECT type E3s. Similar to other NEDD4 family members (nine in total), Smurfs contain: (i) the N-terminal C2 domain, which mediates binding of these E3s to intracellular membranes; (ii) several tryptophan-containing WW domains, which are thought to mediate the protein-protein interactions between the E3s and their interactors and substrates (primarily through association with proline-containing PPxY or LPxY motifs in the binding partners); and (iii) the evolutionary-conserved catalytic HECT domain. Of note, several studies indicate that the HECT domain of NEDD4 E3s are also involved in substrate recognition (5–7).

**Figure 1:**
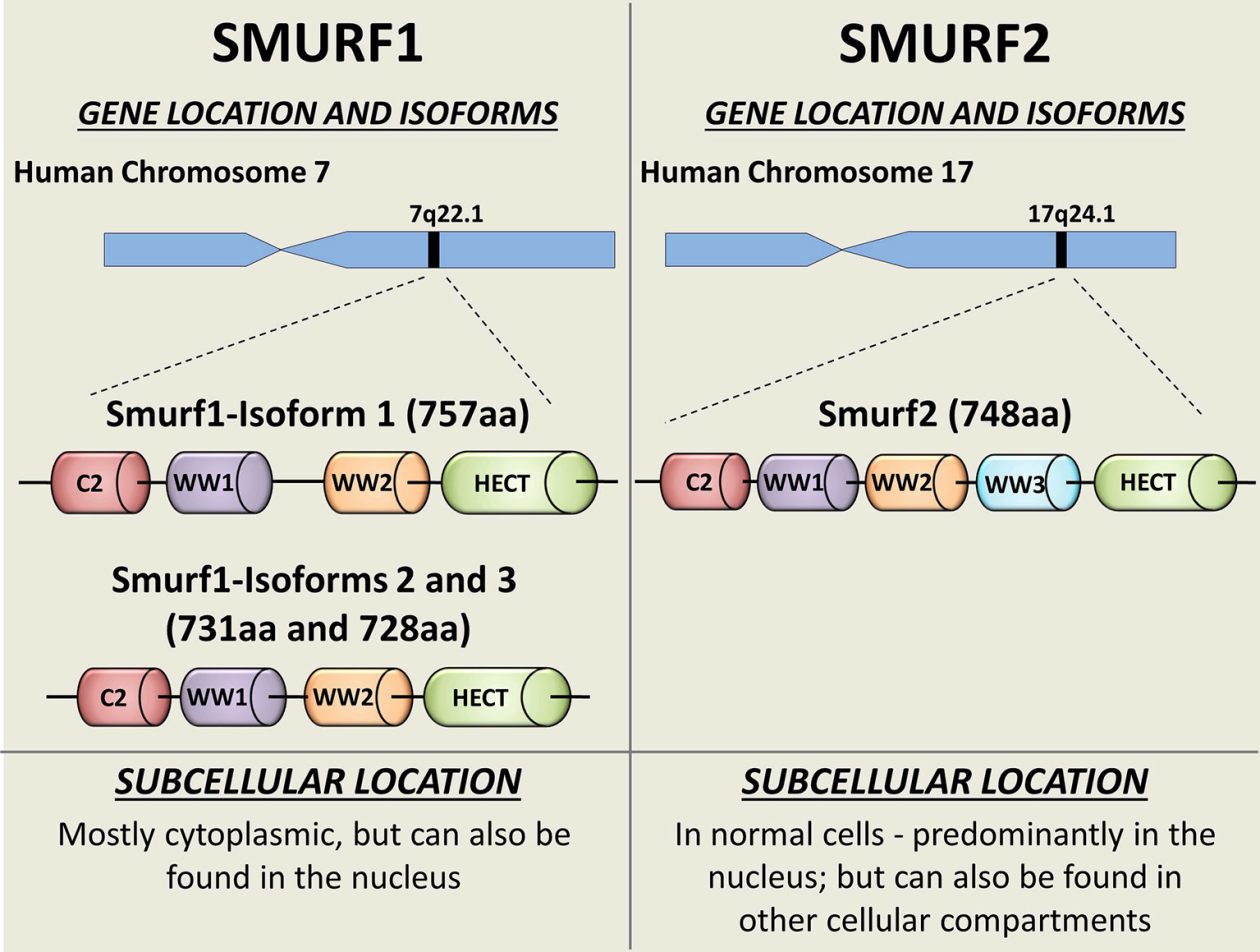
Schematic diagram showing the SMURFs' gene locations, isoforms, and intracellular distribution. Both Smurf1 and Smurf2 possess an N-terminal protein kinase C (PKC)-related C2 domain (Red), 2-3 WW protein interacting domains (purple, orange, and blue) and the catalytical C-terminal HECT domain (green). Smurf1 transcript variant 1 (NP_065162) is the longest Smurf1 isoform (757 aa), and has a 26-residue linker insert between WW domains (8). Catalytical active site—Cys699. Smurf1 transcript variant 2 (NP_851994; 731 aa) lacks an in-frame exon in the coding region, compared to variant 1. Smurf1 isoform 3 (NP_001186776) lacks an in-frame exon in the coding region and uses an alternate in-frame splice site in the 3′ coding region, compared to variant 1. This variant is 728 aa long. Smurf2 (NP_073576) is a 748 aa protein. Catalytical active site—Cys716. Subcellular bio-distributions of Smurf1 and Smurf2 are indicated on the bottom of the figure.

In mammals, Smurf1 and Smurf2 are encoded by two distinct genes located at chromosomes 7 and 17, respectively (Figure 1). Three isoforms of human Smurf1, resulting from alternative splicing, have been reported, and a single protein product has been confirmed for Smurf2 (9).

Smurfs share a high sequence homology (>70% amino acid sequence identity) and have similar structural characteristics. Despite these high similarities and some redundancy in their substrate repertoire, these proteins exhibit distinct and in some respects opposite biological functions.

In this review, we will discuss the diverse roles of Smurf proteins in pleotropic cellular functions, including cell proliferation and DNA damage response, chromatin organization, dynamics and genomic integrity maintenance, gene expression, carcinogenesis, and metastases. We also highlight studies implicating Smurfs in cellular responses to anticancer therapies.

## Regulatory roles of smurfs in the decisive cellular processes

### Smurfs in TGF-β/BMP signaling

Smurf1 and Smurf2 were originally identified as negative regulators of BMP/TGF-β signaling pathways. These pathways play crucial roles in embryogenesis and adult tissue homeostasis, as well as in the pathogenesis of various human diseases (10, 11).

In cancer, these pathways appear to have a dual role: operating in both cancer development and suppression (12, 13). Indeed, the activation and/or inhibition of these pathways are highly related to various aspects of carcinogenesis including epithelial-mesenchymal transition (EMT), angiogenesis, behavior of cancer stem cells, metastases, and tumor cell chemo-refractoriness.

For example, TGF-β signaling exerts in normal cells and at the early-stages of carcinogenesis tumor-suppressor functions, including cell-cycle arrest and triggering of apoptosis. However, at late-stages of carcinogenesis, the role is reversed and TGF-β signaling promotes tumorigenesis, metastases and chemoresistance (13).

Smurf1 has been shown to ubiquitinate and degrade the BMP receptor*-*regulated Smad proteins (R-Smads; i.e., Smad1 and Smad5), which form heteromeric complexes with a common-partner Smad (Co-Smad) Smad4. Following formation, this complex translocates into the nucleus to regulate transcription of a variety of target genes, also related to tumorigenesis, cancer progression, and chemoresistance. The ability of Smurf1 to degrade the BMP-specific Smads as well as BMP receptors, provides negative feedback to the BMP signaling pathway. Noteworthy, Smurf1 can cooperate with inhibitory Smad (I-Smad), Smad6, and Smad7, which repress the TGF-β superfamily signaling by several different mechanisms (14–17).

The E3 ubiquitin ligase functions of Smurf2 were primarily associated with its ability to negatively regulate the TGF-β signaling pathway. Following receptor stimulation with the TGF-β ligand, Smurf2 translocates from the nucleus to the cytosol. For this to occur, nuclear Smurf2 needs to bind to I-Smad, in particular of Smad7, which facilitates the nuclear export of Smurf2.

While in the cytosol, Smurf2 interacts with and promotes the proteasomal degradation of the TGF-β receptor (i.e., TGF-βRI) as well as TGF-β-specific R-Smads such as Smad2 and Smad3 (18–21). Of note, although the Smurf1 and Smurf2 activities are primarily attributed to BMP and TGF-β signaling regulation, respectively, experimental data suggest a model where Smurfs can truncate both of these pathways.

Despite these proceedings, studies conducted on Smurf1- and Smurf2-genetically ablated mice question the role of Smurfs in the *canonical* TGF-β superfamily signaling. *Smurf1* knockout mice revealed no significant disruption in the Smad-mediated TGF-β/BMP signaling pathways. Instead, these animals exhibited an age-dependent increase of bone mass due to enhanced osteoblast activity. This activity was related to activation of the MEKK2-JNK signaling cascade (22).

Targeted disruption of *Smurf2* in mice has also revealed that the protein levels and stability of the TGF-β receptor and Smad proteins (i.e., Smad2/3) were unaffected by *Smurf2* depletion, despite the enhanced cellular response to TGF-β stimulation (23). This phenomenon was explained by the uncovered ability of Smurf2 to monoubiquitinate Smad3 and inhibit the formation of Smad3 complexes (23). The activity of these complexes is required for the TGF-β-mediated transcriptional response.

Collectively, these findings stipulate that at least in mouse experimental models, Smurf proteins do not directly regulate the stability and turnover of the BMP/TGF-β receptors and Smad transducers. Of note, both murine and human Smurf proteins share a very high homology and amino acid identity: 95% for Smurf1 and 99% for Smurf2, suggesting that mouse models can appropriately investigate the biological roles of Smurfs in humans.

In addition, experimental evidence shows that Smurfs are not the only E3 ubiquitin ligases regulating TGF-β signaling. Other NEDD4 E3 ubiquitin ligase family members, including ITCH, NEDD2L, WWP1 and WWP2, can also mitigate this cascade (7). These findings suggest that NEDD4 E3s have overlapping functions in the TGF-β signaling regulation. Moreover, Smurf2 has been reported to ubiquitinate and promote the degradation of Smurf1, introducing further complexities in TGF-β/BMP signaling regulation by Smurfs (24).

Interestingly, a recent study shows that mice deficient for *Smurf2* exhibit decreased bone mass due to severe osteoporosis. This phenotype is opposite to the phenotype observed in *Smurf1*-ablated animals (25). Moreover, the authors demonstrate that elimination of Smurf2, but not Smurf1, significantly increases the expression of RANKL, a key regulator in osteoclastogenesis and bone physiology. Through mechanistic studies, they showed that this phenomenon is associated with the ability of Smurf2 to monoubiquitinate and inactivate transcriptional activity of Smad3, as previously reported (23). In the absence of Smurf2, Smad3 activity remains unrestrained and results in enhanced transactivation activity of the vitamin D receptor (VDR) signaling, ultimately leading to elevated expression of RANKL.

### Smurfs in carcinogenesis

#### Smurf2 acts as a tumor suppressor

The most surprising and exciting finding on the involvement of Smurfs, in particular of Smurf2, in cancer we, and subsequently another group, obtained using Smurf2-depleted mice (*Smurf2*^−/−^ mice). We found that while relatively normal in their early lives, these mice developed a wide spectrum of tumors in different organs and tissues as they aged. The majority of tumors appeared in mice older than 90 weeks (7, 26), equivalent to ~70 years of age in humans (27). About 70% of the uncovered tumors were of epithelial origin including hepatocellular carcinoma, lung alveolar carcinoma, mammary gland carcinoma, and others. Hematological malignancies were also detected, in ~30% of the cases.

To best of our knowledge, there are only a few, if any, mouse cancer models which so closely mimic two key characteristics of human cancer: (i) the late cancer onset (~77% of all cancers are diagnosed in persons 55 years of age and older); and (ii) the epithelial origin of tumors. For example, the vast majority of tumors in *p53*-null mice are hematological malignancies (mostly lymphomas), which develop within a few months after the animal's birth (28, 29).

These findings suggest that the *Smurf2*-ablated animals are highly relevant to human carcinogenesis model, and could be advantageous when studying cancer-related processes at the whole organism level.

Moreover, Zhang's group further demonstrated that mice heterozygote for Smurf2 (*Smurf2*^+/−^) are also susceptible to spontaneous tumorigenesis (30). Further analysis of tumors from these animals revealed the loss of heterozygosity (LOH) at *Smurf2*. LOH is a common genetic event inactivating residual wild type allele of genes, in particular of tumor suppressors.

Altogether, these findings establish Smurf2 as a potent tumor suppressor, preventing the transformation of normal cells into cancerous ones.

#### Smurf2 regulates chromatin organization, dynamics, and integrity

**Figure 2:**
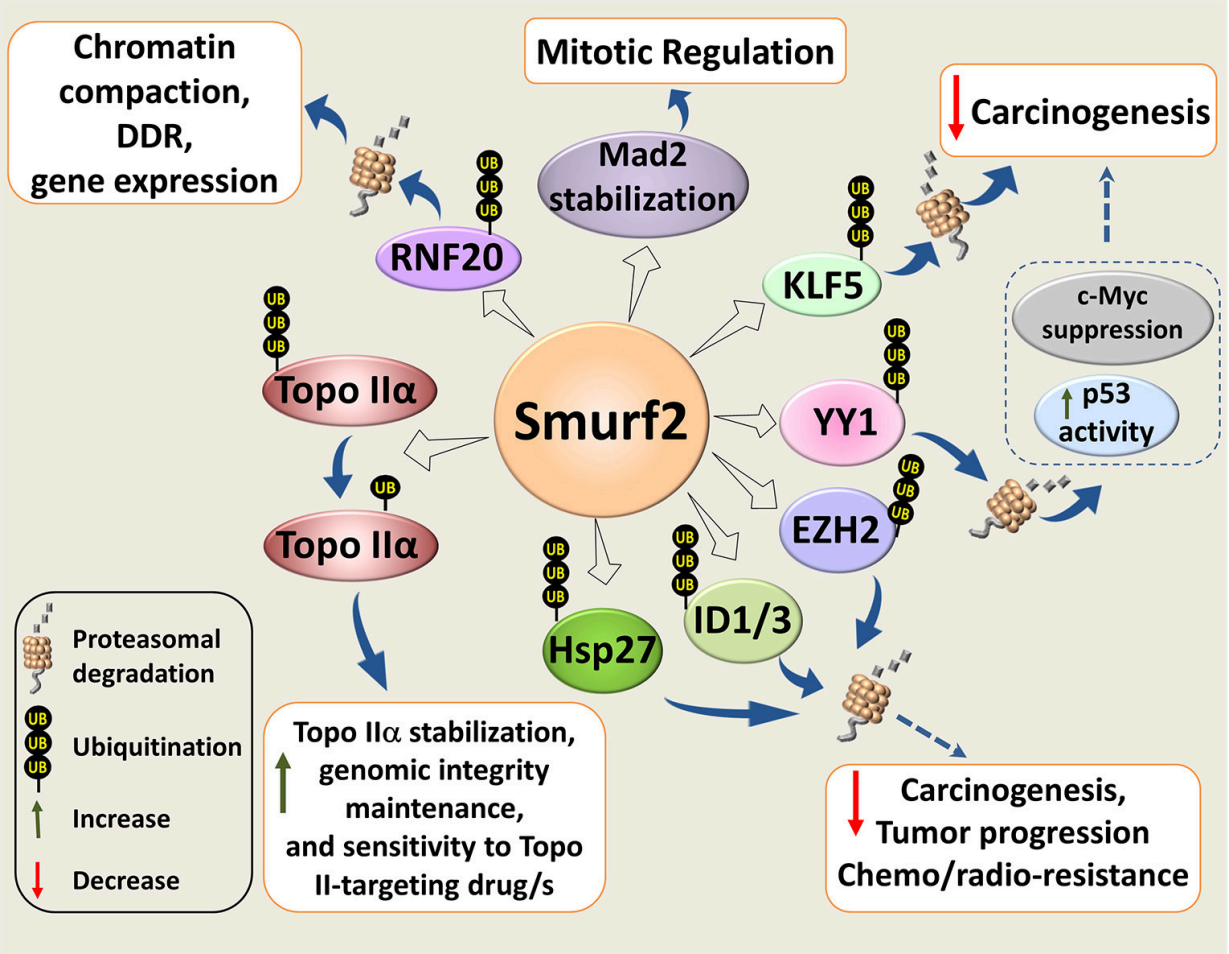
Molecular mechanisms underlying Smurf2 tumor suppressor functions. Smurf2 regulates chromatin compaction, DDR, and gene expression through the ubiquitin-proteasomal degradation of RING-type E3 ubiquitin ligase and histone H2B modifier RNF20. Smurf2 also controls chromatin organization, dynamics and unaltered chromosomal inheritance through stability regulation of Topo IIα. In addition, Smurf2 could affect carcinogenesis, tumor progression and sensitivity to anticancer therapies through the ubiquitin-mediated proteasomal turnover of KLF5, ID1/3, YY1, and others. Degradation of YY1, for example, relieves the suppression of p53 activity by YY1, and decreases the expression of c-Myc. Smurf2-mediated stabilization of the mitotic spindle checkpoint protein Mad2, is also shown in the diagram. Dotted arrows specify potential mechanisms determined from existing evidence.

Our subsequent studies revealed that inactivation of Smurf2 triggers a series of cascading events in cells, and creates the “mutator phenotype,” which under the stress of aging leads to carcinogenesis (26). Mechanistically, we found that Smurf2 regulates chromatin structure landscape and, thereby, affects gene expression, DNA damage response (DDR), and genomic integrity maintenance. We further demonstrated that these Smurf2 activities were associated with and at least in part relied on its ability to ubiquitinate and degrade RNF20 (Figure 2), a RING type E3 ubiquitin ligase responsible for monoubiquitination of histone H2B (ubH2B). The RNF20-ubH2B module regulates chromatin compaction, DNA damage response, and gene expression, and acts both as a tumor suppressor and an oncogene depending on the cellular context (26, 31–39).

Furthermore, an interesting finding in *Smurf2*^−/−^ cell genome was the accumulation of multiple chromosomal abnormalities, with translocations being the most notable hallmark (26). Subsequent investigation of this phenomenon showed that Smurf2 expression is essential in preventing the formation of pathological chromatin bridges, also known as anaphase bridges (40). These bridges are a major cause of chromosomal translocations, and are often an output of the compromised decatenation checkpoint.

The decatenation checkpoint is normally mediated by DNA topoisomerase IIα (Topo IIα), a core enzyme in chromatin organization, dynamics and unaltered chromosome inheritance (41).

We found that Smurf2 operates as a molecular editor of Topo IIα, switching its ubiquitination code from the degradation-promoting K48 polyubiquitination to monoubiquitination, and stabilizing the enzyme (40) (Figure 2). Unaltered E3 ubiquitin ligase functions of Smurf2 were indispensable for this regulation. Moreover, we showed that Smurf2 depletion phenocopied Topo IIα depletion and increased the formation of anaphase bridges. Introduction of Topo IIα into Smurf2-depleted cells rescued this phenomenon. Our studies also uncovered that Smurf2 is a determinant of Topo IIα protein levels in cancer cells and tissues, and is a factor affecting tumor cell sensitivity to the Topo II-targeting drug, etoposide (40).

Collectively, these findings establish Smurf2 as a key cellular factor governing chromatin organization, dynamics and genome integrity maintenance. They also indicate Smurf2 as a potent tumor suppressor.

#### Other putative mechanisms for smurf2-mediated tumor suppression

In addition to RNF20 and Topo IIα, Smurf2 has been shown to regulate stability and/or subcellular localization of other decisive cellular proteins implicated in carcinogenesis and drug resistance. These molecules include the molecular chaperone and apoptosis inhibitor HSP27, transcription factors KLF5, YY1, ID1/ID3, histone methyltransferase EZH2, and others (Figure 2).

HSP27 (heat shock protein 27) is one of the central molecules shown to upregulate EMT and affect activities of the matrix metalloproteinases (MMPs), stimulate tumor cell proliferation, migration, invasion, as well as to mediate chemo- and radio-resistance (42). Smurf2 overexpression was reported to alter HSP27 subcellular distribution and induce its ubiquitin-dependent degradation in the human lung adenocarcinoma A549 cell model (43). However, it currently remains unknown whether these Smurf2 activities are pertinent to its tumor suppressor functions in lung cancer, and/or in other types of tumors.

Smurf2 was also shown to promote the degradation of a few principal transcription factors whose activities are associated with carcinogenesis, drug resistance, patient prognosis and survival. Krüppel-like factor 5 (KLF5) is one of these factors with cell growth-promoting and pro-survival activities. KLF5 is also implicated in cell differentiation, migration and stemness and its expression levels are frequently abnormal in different types of cancer (44). Smurf2 was shown to polyubiquitinate and promote the proteasomal degradation of KLF5 in a Smurf2 E3 ligase-dependent manner, thereby inhibiting the transcriptional and pro-proliferative activities of KLF5 (45). Interestingly, KLF5 levels were specifically reduced by Smurf2, but not by Smurf1.

Yin Yang 1 (YY1) is another example of the Krüppel-like zinc finger transcriptional factors negatively regulated by Smurf2. YY1 is overexpressed in multiple cancer types, and its overexpression correlates with poor clinical outcomes, although several studies suggested that in some types of cancer YY1 acts as a tumor suppressor (46). Two research groups reported that Smurf2 ubiquitinates and promotes the degradation of YY1 (47, 48). The outcomes of these Smurf2-mediated effects were a decrease in the YY1-mediated suppression of p53 activity (47), and a reduction of B-cell proliferation and lymphomagenesis (48). The latter was supposedly mediated via the suppression of YY1-c-Myc regulatory axis (Figure 2).

The ability of Smurf2 to ubiquitinate and degrade two dominant inhibitors of helix-loop-helix transcription factors ID1 and ID3 (49) might also be relevant to Smurf2's tumor suppressor activities. Overexpression of these IDs was shown to facilitate tumor growth, angiogenesis, stem cell maintenance, invasiveness, metastasis, as well as correlating with unfavorable clinical prognoses (50, 51). Moreover, ID1 has been shown to confer chemoresistance to different types of cancer (52–55).

In addition, Smurf2 was shown to polyubiquitinate and induce a proteasome-mediated degradation of EZH2, the catalytic subunit of the polycomb repressive complex 2 (PRC2) and histone H3-K27 methyltransferase (56). This was reported in human mesenchymal stem cells during their neuronal differentiation. If this finding is corroborated in tumor cell models it might be highly pertinent to the ability of Smurf2 to interfere with carcinogenesis, as EZH2 was documented as a pro-oncogenic factor involved in neoplastic transformation, cancer cell stemness, metastases and immune evasion. However, it should be mentioned that several studies show that under some circumstances EZH2 also exhibits tumor suppressive activities (57).

Smurf2 has also been implicated in the formation of the functional mitotic spindle checkpoint by regulating the localization and stability of the MAD2 protein (58). Knockdown of Smurf2 or overexpression of its E3 ligase-deficient mutant generated misaligned and lagging chromosomes, premature anaphase onset, and defective cytokinesis in human cervix carcinoma HeLa cells (58). Interestingly, in our study, Smurf2 depletion did not affect the formation of lagging chromosomes, but instead increased the formation of anaphase bridges in osteosarcoma U2OS cells (40). These discrepancies could be explained by different types of cancer cell models used in these studies: HeLa vs. U2OS cells, implying that biological effects of Smurf2 should be very carefully interpreted taking into account cellular context, genetic make-up, and experimental settings.

Altogether, these findings designate Smurf2 as a pleotropic cellular factor that regulates a wide spectrum of molecular pathways and networks to control transcription, DNA damage response and genomic integrity maintenance. When these pathways are compromised, carcinogenic processes can be set in motion, leading to cell transformation and the development of a wide spectrum of tumors, as observed in *Smurf2*-null mice (7, 26).

Furthermore, the Smurf2-Smad3-RANKL axis described in the previous section could also potentially be involved in tumor formation in Smurf2-deficient animals. This appears to be most relevant to mammary gland carcinomas developed in *Smurf2*^−/−^ mice, as the upregulated RANKL/RANK signaling pathway could promote mammary stem cell expansion, proliferation and the formation of hormone-induced breast cancer (59).

Interestingly, genomic studies showed that the SMURF2 gene is not frequently mutated in human malignancies (https://cancer.sanger.ac.uk/cosmic/gene/analysis?ln=SMURF2). However, changes in Smurf2 expression are common in many cancers (7, 26, 40), similar to some other cancer-related genes such as the two TP53 paralogs, TP63 and TP73 (60), and the members of the FOXO transcription factors family (61).

#### The duality of smurf2 in cancer

As described above, evidence points to Smurf2 as a potent tumor suppressor operating in *normal* cells to prevent cell transformation and carcinogenesis. However, results obtained in established cancer cell models argue that Smurf2 has a dual role and under some circumstances acts as an oncogene rather than a tumor suppressor (7, 9, 62). Additionally, the expression levels of Smurf2 were reported to be significantly elevated in several types of cancers including esophageal squamous cell carcinoma tumors (63) and chemo-refractory tumors such as recurrent hepatocellular carcinomas (64). The data available in the COSMIC dataset portal also indicate that Smurf2 is overexpressed in ~49% of ovarian cancer, about 18% of breast cancer, and in ~17% of soft tissue neoplasms.

Furthermore, ours and other studies show that subcellular biodistribution of Smurf2 is prominently altered in cancer *vs*. normal cells, with a notable accumulation/sequestration of Smurf2 in the cytoplasm of tumor cells (7, 26, 65). It is possible that overexpressed and mislocalized Smurf2 is employed by the carcinogenic machinery to promote oncogenesis, at least in some types of cancer.

**Figure 3:**
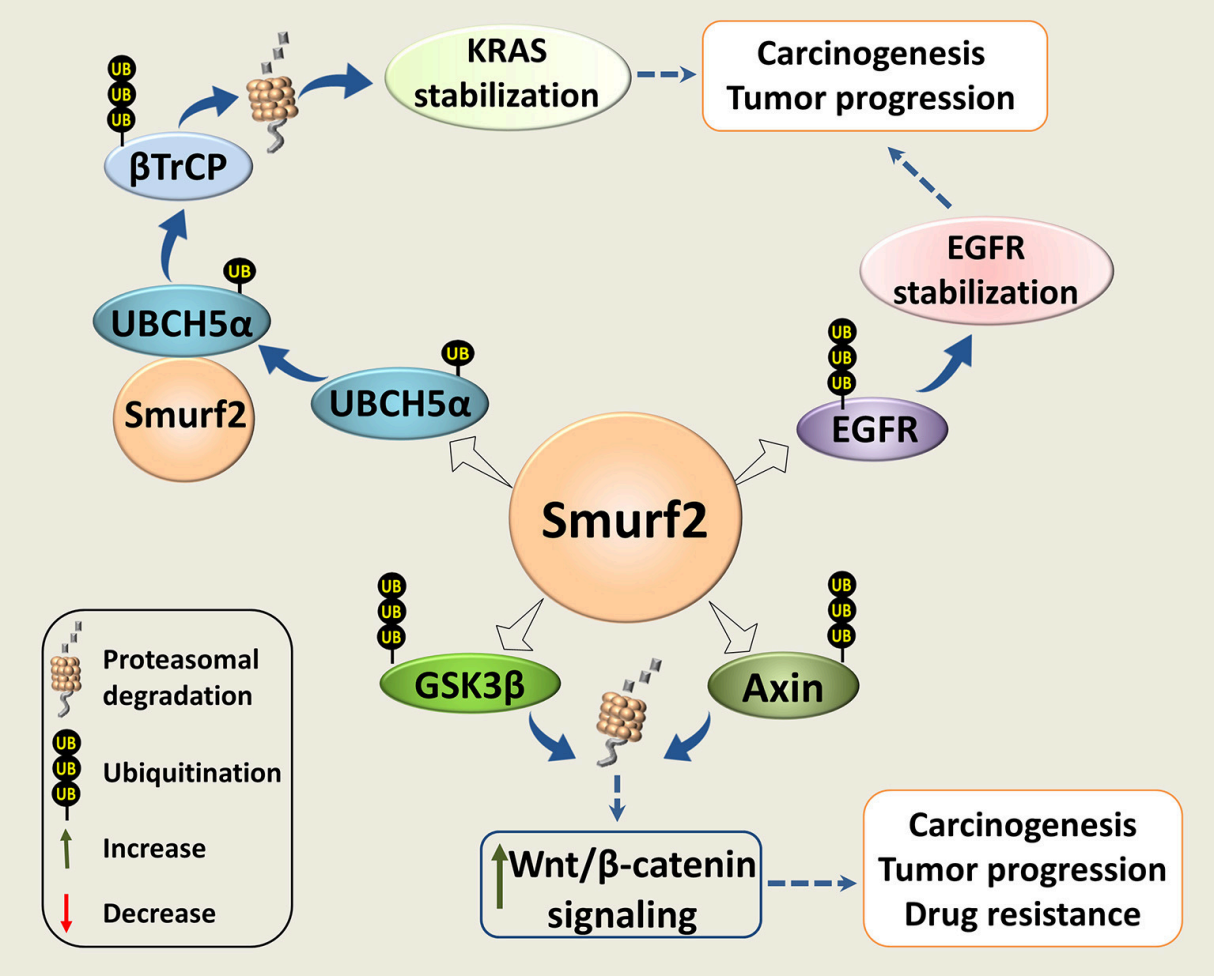
Smurf2 as an oncogene. Smurf2 monoubiquitinates UBCH5α and forms an active complex for the degradation of the KRAS negative regulator, βTrCP. This stabilizes KRAS and could potentiate its pro-oncogenic functions. Smurf2 also ubiquitinates EGFR and protects it from degradation. In addition, Smurf2 through the degradation of GSK3β and Axin could upregulate the Wnt/β-catenin signaling. Through this mechanism, Smurf2 might also increase the pro-oncogenic activities of this pathway. Dotted arrows specify potential mechanisms determined from existing evidence.

The possible pro-oncogenic functions of Smurf2 in genetically-compromised tumor cells could be related to the reported ability of Smurf2 to interfere with the RAS, Wnt/β-catenin, and EGFR-mediated signaling pathways, three central modules in cancer progression and chemoresistance (Figure 3).

It has been reported that Smurf2 together with the E2 ubiquitin-conjugating enzyme UBCH5, stabilize the KRAS oncoprotein (66), the most frequently mutated transforming oncogene in human cancers (67). The authors showed that Smurf2 monoubiquitinates UBCH5 to form an active complex for degradation of β-TrCP, the F-box protein and a component of SCF E3 ligase that negatively regulates KRAS (68). Loss of Smurf2 led to the accumulation of β-TrCP, and KRAS degradation. Interestingly, silencing of Smurf2 mostly affected the mutant form/s of KRAS. In addition, the authors demonstrated that knockdown of Smurf2 reduces the clonogenic survival and prolongs tumor latency in the mutant KRAS-driven tumors generated in nude mice with either human colon or lung carcinoma cells (66).

Under the described experimental setting, Smurf2 appears to act as an oncogene, promoting tumor development. However, it should be mentioned that the SCF^β−*TrCP*^ complex mediates the degradation of functionally diverse proteins, and it is capable to downregulate both oncogenes (i.e., RAS, β-catenin, CDC25A, and others) as well as tumor suppressors (e.g., Smad4, IkB, FOXO3 and REST) (69, 70). Thus, the role of the Smurf2/SCF^β−*TrCP*^ module in cancer may vary considerably depending on the cell type and molecular composition, and should be determined in a particular context.

Smurf2 was also reported to promote the Wnt/β-catenin signaling through the degradation of its two negative regulators: GSK3β (71) and Axin (72). Through this route, Smurf2 could potentially facilitate the activities of the proto*-*oncogene β*-*catenin.

Interestingly, GSK3β phosphorylates and primes RAS proteins for SCF^β−*TrCP*^-mediated degradation (73). Inhibition of this degradation pathway by aberrant Wnt/β-catenin signaling may contribute to Ras-induced transformation in colorectal tumorigenesis (68). In this regard, it will be even more important to investigate the role of the Smurf2/GSK3β/SCF^β−*TrCP*^/Ras module in animal cancer models and in clinical samples.

Smurf2 has also been shown to ubiquitinate and protect from c-Cbl-mediated degradation the epidermal growth factor receptor (EGFR), which is implicated in a wide range of cell responses ranging from cell division to adhesion, motility, and death (74). The authors also reported that the loss of Smurf2 destabilizes EGFR, and reduces the clonogenic survival of EGFR-expressing cancer cell strains. The effects of Smurf2 depletion on EGFR-negative cancer cells, normal fibroblasts, and on normal epithelial cells were minor. In addition, the authors demonstrated that knockdown of Smurf2 reduces the ability of human head and neck squamous cell carcinoma UMSCC74B cells to form tumors *in vivo*.

Cooperatively, these studies suggest that in immortalized and established cancer cell models Smurf2 operates as an oncogene rather than a tumor suppressor. Of note, Wellbrock's group reported that Smurf2 depletion can significantly increase melanoma cell sensitivity to the cytotoxic effects of the MEK inhibitor selumetinib (AZD6244), both *in vitro* and *in vivo* (75).

This finding implies that at least in this type of tumor, inactivating Smurf2 might overcome tumor cell resistance to MAPK pathway inhibitors experienced in clinics.

### Smurfs in tumor cell proliferation, migration, and invasion

A few studies have shown that Smurf2 is intrinsically involved in these critical for cancer progression processes. However, studies from different groups revealed different and in some respect contradictory results, even when using the same cancer cell model. For example, Zhang's group demonstrated that elevated levels of Smurf2 were required for and promoted migration, invasion and *in vivo* metastatic dissemination of human breast carcinoma MDA-MB-231 cells. Moreover, the authors demonstrated that Smurf2 E3 ligase-defective mutant (Cys716Gly) decreases the metastatic behavior of these cells (76).

In contrast, Imamura's group showed that Smurf2 knockdown in MDA-MB-231 cells enhances cell migration *in vitro* and bone metastasis *in vivo*, implying that under these circumstances Smurf2 is a tumor suppressor (24). The same group also demonstrated that Smurf2 reduces MDA-MB-231 cell migration via Smurf1 degradation. The authors also provided evidence that the motility of Smurf2-knocked down cells is independent of TGF-β-signaling.

In addition, a recent study showed that knock-down of Smurf2 increases the proportion of invasive MDA-MB-231 cell-derived organoids. This group also demonstrated that PIAS3-dependent sumoylation of Smurf2 is important in suppressing the invasive behavior of these cells (77).

The discrepancies in these studies could be explained, at least in part, by different approaches used to manipulate the Smurf2 expression levels (overexpression *vs*. knock-down). However, a more comprehensive investigation is needed to support this notion. One possible approach is to examine these effects in SMURF2 genetically-ablated MDA-MB-231 cells, as well as in other human cancer cell models. These cells were recently generated by our group using the CRISP/Cas9 gene-editing tool (78), and are currently under investigation.

Studies conducted using pancreatic cancer cells also suggest that Smurf2 acts as a tumor suppressor. The authors showed that Smurf2 is downregulated in pancreatic cancer tissues, and its overexpression suppresses migration and invasion of pancreatic cancer cells, while having no effect on cell viability, cell cycle, and senescence (79). Interestingly, the authors also showed that Smurf2 promotes mesenchymal-epithelial transition (MET), and that its expression levels are negatively associated with cancer cell resistance to gemcitabine treatment.

**Figure 4:**
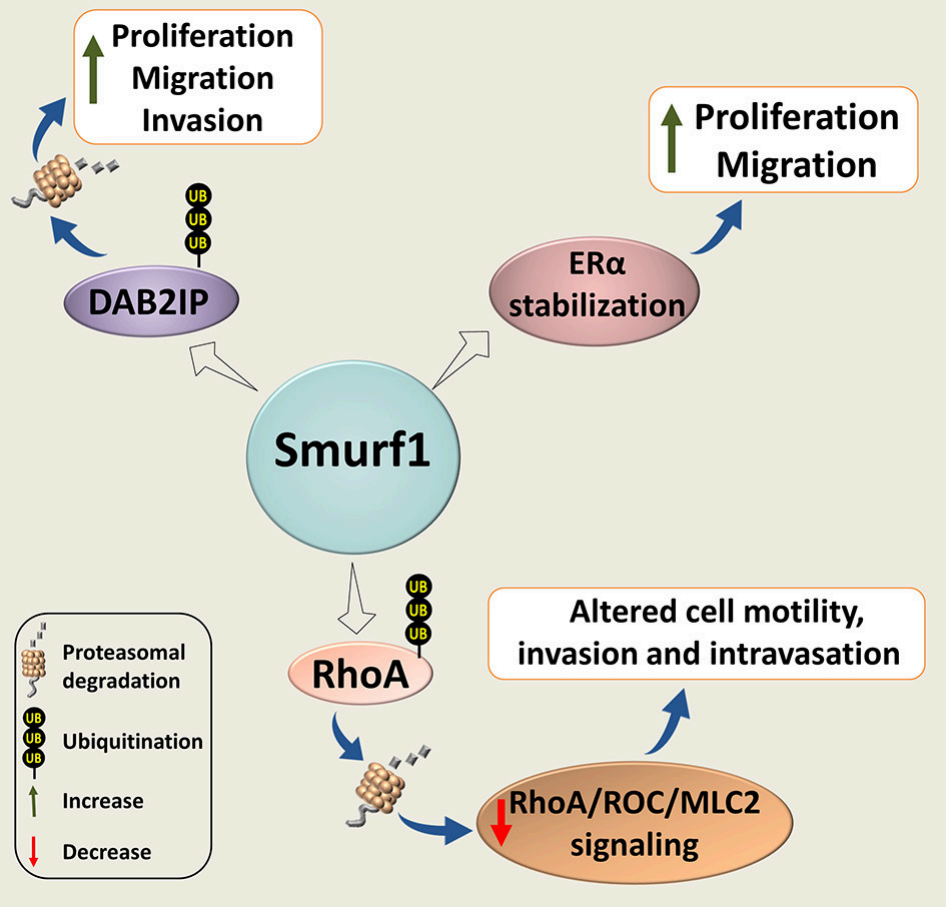
Smurf1 in cancer cell proliferation, motility, and invasion. Smurf1 promotes ubiquitin-dependent degradation of DAB2IP; and stabilizes ERα, resulting in accelerating tumor cell replication, migration and invasion. Smurf1 can also affect cancer cell motility and invasion by proteasomal degradation of RhoA.

Smurf1 has also been implicated in cancer cell proliferation, migration and invasion (Figure 4). Wrana's group found that Smurf1 plays an important role in regulating protrusive activity and the transformed phenotype of HEK293T cells (80). Mechanistically, the authors demonstrated that Smurf1 is recruited by PKCζ to cellular protrusions, where it controls the protein levels of RhoA, a small GTPase implicated in cell shape, polarity, adhesion, and motility regulation. Smurf2 however, was not involved in the RhoA stability regulation.

Subsequently, Vial's group showed that Smurf1 expression is required for lamellipodia formation, tumor cell plasticity, and motility through the regulation of peripheral RhoA/ROCK/MLC2 signaling. Silencing of Smurf1 or expression of its interfering mutants inhibited cell migration (81). Interestingly, their *in vivo* studies showed that Smurf1 reduction induces the mesenchymal-amoeboid transition, facilitates cell motility and increases invasion and intravasation. However, this reduction was insufficient to promote metastasis after cells have entered the vessels.

In another study, induction of Smurf1 expression either by EGF or by overexpression of MEK1, as well as Smurf1 overexpression, significantly increased migration and invasion of breast carcinoma MDA-MB-231 cells, whereas knockdown of Smurf1 suppressed the phenotype (82). These findings are in agreement with results reported by Imamura's group (24).

The pro-oncogenic role of Smurf1 was also noted in other types of cancer. For example, suppression of Smurf1 expression in human ovary carcinoma SK-OV-3 and OVCAR-3 cells significantly decreases cell migration and invasion (83). Similar results were also observed in prostate cancer cell models (84). In addition, the authors demonstrated that expression of Smurf1 in prostate cancer cells is regulated through the androgen receptor (AR) signaling, which is critical for prostate cancer growth and survival.

Another recently published study shows that Smurf1 expression is also triggered through estrogen signaling (85). The authors also demonstrated that Smurf1 stabilizes estrogen receptor alpha (ERα) in breast cancer cells, leading to increased estrogen signaling and enhanced cell proliferation. These findings suggest a forward feedback loop in Smurf1-ERα regulation.

Smurf1 is also overexpressed in human gastric cancer (GC) tissues (86). Moreover, Smurf1 expression levels were shown to be positively associated with more advanced tumor-node-metastasis (TNM) stage of GC, and inversely correlated with patient survival. Knockdown of Smurf1 inhibited proliferation, migration and invasion of GC cells, at least in some GC cell models, while Smurf1 overexpression exacerbated these phenotypes. Furthermore, the authors reported that Smurf1-knockdown in GC cells markedly inhibits tumor growth and liver metastasis *in vivo*. Mechanistically, they linked the Smurf1 pro-oncogenic activities with the ability of Smurf1 to negatively regulate the expression of DAB2IP, a GTPase-activating protein (GAP) and a suggested tumor suppressor (86).

The existence of similar Smurf1-regulated tumor promoting mechanism was also observed in clear cell renal cell carcinoma (ccRCC) cells (87). This mechanism was associated with the ability of Smurf1 to promote proliferation, migration, and invasion of ccRCC cells. In addition, the expression levels of Smurf1 were found to be elevated both in ccRCC cell lines and cancer tissues, and associated with worse patient survival.

## Concluding remarks and future directions

In this review, we highlighted and discussed the cancer related biological functions of two C2-WW-HECT E3 ligases, Smurf1 and Smurf2. These proteins surfaced as influential, and under some circumstances, as decisive cellular factors regulating a plethora of cellular processes pertinent to cancer onset, progression and therapy.

The currently available data stipulate that Smurf1 acts as an oncogene, whereas Smurf2 operates both as a tumor suppressor and a tumor promoting molecule, depending on the tumor stage, type, molecular binding partners, and other still unidentified factors.

It is now evident that apart from TGF-β/BMP signaling, Smurfs regulate different signaling pathways and networks. Understanding these networks and the Smurfs' impact on their components is important and should be further investigated.

Another line of investigation is elucidating the mechanisms regulating expression, localization and functions of Smurfs. Currently, these mechanisms remain elusive. Understanding of these mechanisms is imperative in explaining the dual role of Smurf2 in cancer and its impact on cancer progression and treatment. In addition, the full spectrum of mechanisms and networks operating under Smurf1/2 auspices is also currently unknown.

**Figure 5:**
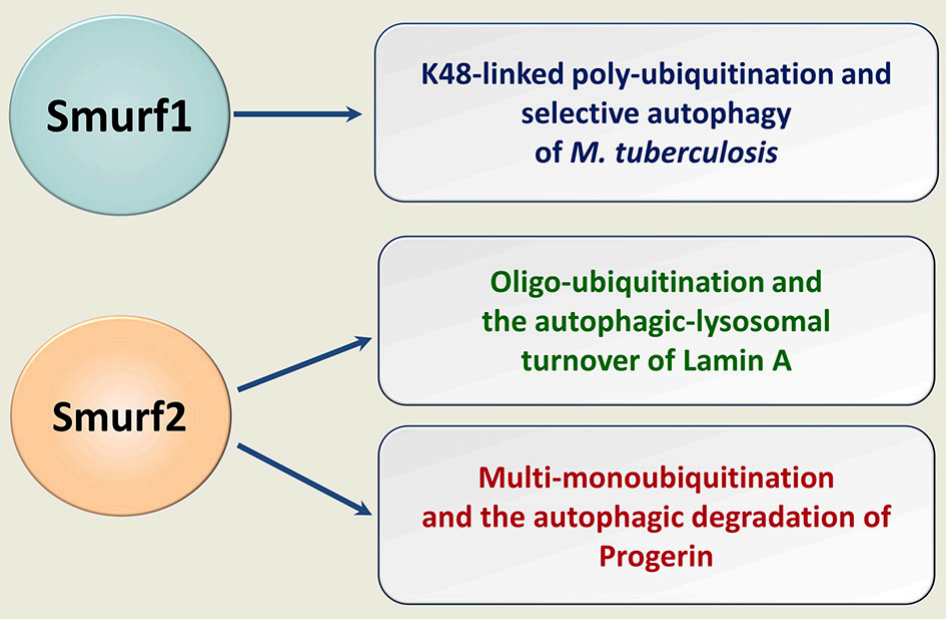
Smurfs in selective autophagy of human pathogens and endogenous cellular proteins. Smurf1 mediates the K48-linked polyubiquitination of human pathogen *M. tuberculosis*, targeting it for the ubiquitin-dependent selective autophagy. Smurf2 oligo-ubiquitinates lamin A and multi-monoubiquitinates progerin. Smurf2 targets both lamin A and progerin for the autophagic-lysosomal turnover.

Recently, we have shown that Smurf2 in addition to its ability to control protein homeostasis through the proteasomal breakdown, targets some cellular proteins for autophagic-lysosomal turnover (88). Specifically, we found that Smurf2 regulates selective autophagy of nuclear lamins, in particular lamin A, and its mutant form progerin (Figure 5). The expression of progerin underlies the pathogenesis of the devastating premature aging syndrome, HGPS (Hutchinson-Gilford progeria syndrome). Remarkable, in addition to HGPS, progerin also accumulates in cells during physiological aging and supposedly in cancer, where it could promote genomic instability and increase tumorigenesis (89–91). This association suggests that targeting progerin through the Smurf2-mediated autophagy might be a promising direction to eradicate tumor cells, though more research is needed in this regard.

Smurf1 has also been implicated in selective autophagy, in particular in eliminating in human cells of Mycobacterium tuberculosis (92) (Figure 5). Whether Smurf1 can also mediate the autophagic degradation of endogenous cellular proteins is currently unknown.

Both the ubiquitin-proteasome and autophagic-lysosomal degradation pathways are intrinsically involved in cancer initiation, progression and cure. Thus, understanding mechanisms operating in the intersection of these protein turnover machineries could provide novel therapeutic targets and paradigms in cancer diagnosis and treatment. Further efforts should be directed to characterize the involvement of Smurf proteins in these processes. Another important direction that we believe should be investigated is elucidating whether and how targeting of Smurfs by pharmacological intervention (e.g., by Smurf catalytic inhibitors) affect the ability of tumor cells to escape the destructive impact of anticancer drugs and therapies used in clinics. This is a more long-term goal as such compounds are currently unavailable.

## Author contributions

PK and GL-C drafted the initial version of the manuscript, and prepared figures and bibliography. MB conceptualized the study and wrote the manuscript.

### Conflict of interest statement

The authors declare that the research was conducted in the absence of any commercial or financial relationships that could be construed as a potential conflict of interest.

## Abbreviations

aa: amino acids
AR: Androgen receptor
BMP: Bone morphogenetic protein
BMPRI, Bone morphogenetic protein receptor: type 1
β-TrCP: β-transducing repeat containing protein
ccRCC: Clear cell renal cell carcinoma
CDC25A: Cell division Cycle 25A
COSMIC: Catalog of Somatic Mutations in Cancer
Cys: Cystein
DAB2IP: Disabled homolog 2-Interacting Protein
DDR: DNA damage response
E3s: E3 ubiquitin ligases
EGFR: Epidermal growth factor receptor
EMT: Epithelial-mesenchymal transition
ERα: Estrogen receptor alpha
EZH2: Enhancer of zeste homolog 2
GAP: GTPase-activating protein
GC: Gastric cancer
Gly: Glycine
GSK3β: Glycogen synthase kinase 3 beta
GTPase: Guanosine triphosphatases
H2B: Histone H2B
HECT: Homologous to E6-AP carboxyl terminus
HGPS: Hutchinson-Gilford progeria syndrome
HSP27: Heat shock protein 27
ID1: DNA-binding protein inhibitor ID1
ID3: DNA-binding protein inhibitor ID3
I-Smad: Inhibitory Smads
JNK: c-Jun N-terminal protein kinase
KLF5: Krüppel-like factor 5
KRAS: Kirsten rat sarcoma protein
LOH: Loss of heterozygosity
MAD2: Mitotic spindle assembly checkpoint protein
MAPK: Mitogen-activated protein kinase
MAPK: Mitogen activated protein kinase
MLC2: Myosin light chain 2
MEK: Mitogen-activated protein kinase kinase
MEKK2: Mitogen-activated protein kinase kinase kinase 2
MET: Mesenchymal-epithelial transition
NEDD4: Neural precursor cell expressed developmentally down-regulated protein 4
PIAS3: Protein inhibitor of activated STAT3
PKCζ: Protein kinase C zeta
PRC2: Polycomb repressive complex 2
R-Smad: Receptor regulated Smad
RANKL: Receptor activator of nuclear factor kappa-B ligand
RBR: Ring-in-Between-RING fingers
REST: RE1 silencing transcription factor
RhoA, Ras homolog gene family: member A
RING: Really interesting new gene
RNF20: Ring Finger Protein 20
SCF: Skp1–Cullin–F-box proteins
Smurf: Smad ubiquitin regulatory factor
TGF-β: Transforming growth factor beta
TGF-βRI: Transforming growth factor beta receptor I
TNM: Tumor node metastasis
Topo Iiα: DNA Topoisomerase 2 alpha
ubH2B: monoubiquitinated histone H2B
WWP1: WW domain containing E3 ubiquitin protein ligase 1
WWP2: WW domain containing E3 ubiquitin protein ligase 2
YY1: Yin Yang 1.
